# Molecular regulation after mucosal injury and regeneration in ulcerative colitis

**DOI:** 10.3389/fmolb.2022.996057

**Published:** 2022-10-13

**Authors:** Lie Zheng, Sheng-Lei Duan, Xin-Li Wen, Yan-Cheng Dai

**Affiliations:** ^1^ Department of Gastroenterology, Shaanxi Hospital of Traditional Chinese Medicine, Xi’an, Shaanxi Province, China; ^2^ Department of Gastroenterology, Shanghai Traditional Chinese Medicine Integrated Hospital, Shanghai University of Traditional Chinese Medicine, Shanghai, China

**Keywords:** molecular regulation, mucosal injury, regeneration, ulcerative colitis, intestinal stem cells

## Abstract

Ulcerative colitis (UC) is a chronic nonspecific inflammatory disease with a complex etiology. Intestinal mucosal injury is an important pathological change in individuals with UC. Leucine-rich repeat-containing G protein-coupled receptor 5 (LGR5+) intestinal stem cells (ISCs) exhibit self-renewal and high differentiation potential and play important roles in the repair of intestinal mucosal injury. Moreover, LGR5+ ISCs are intricately regulated by both the Wnt/β-catenin and Notch signaling pathways, which jointly maintain the function of LGR5+ ISCs. Combination therapy targeting multiple signaling pathways and transplantation of LGR5+ ISCs may lead to the development of new clinical therapies for UC.

## Introduction

Ulcerative colitis (UC) is a chronic nonspecific inflammatory disease of the colorectal mucosa that manifests as chronic inflammation and repeated ulceration, seriously affecting the quality of life of patients. The incidence of colorectal cancer is increased in patients with UC over the long course of this disease (Huang et al., 2022; Lavelle et al., 2022; Tian et al., 2022). Intestinal stem cells (ISCs) at the bottom of colonic crypts proliferate and differentiate into mature colonic epithelial cells along the crypt–villus axis, producing progeny that continuously proliferate and eventually reach the top of the colonic crypt, thereby maintaining the renewal, regeneration and repair of damaged intestinal mucosal epithelial cells (Chong et al., 2020; Mishra et al., 2020; Huldani et al., 2022; Serigado et al., 2022). However, the normal and orderly regeneration and repair process is often destroyed or interrupted after mucosal inflammatory injury (Parikh et al., 2019). Therefore, exploring the molecular mechanism regulating intestinal epithelial tissue renewal is helpful for understanding mucosal healing and developing clinical treatments for patients with UC. At present, the specific mechanism involved in maintaining the dynamic balance between UC mucosal inflammatory injury and regeneration and repair is unclear. Leucine-rich repeat-containing G protein-coupled receptor 5 (LGR5) is an important marker of ISCs and is a target in the Wnt/β-catenin pathway (Carmon et al., 2017; Zhang et al., 2021a; Zhu et al., 2021; Zhao et al., 2022). LGR5+ cells are ISCs with multidirectional differentiation potential (Dame et al., 2020; Zhang et al., 2021a). Through the regulation of signaling pathways, LGR5+ ISCs repair the damaged intestinal mucosa and maintain intestinal homeostasis through their self-renewal and differentiation potential. The internal mechanisms through which different signaling pathways interact and synergistically regulate the differentiation of LGR5+ cells in individuals with UC remain unclear (Guiu et al., 2019; Hahn et al., 2019; Yokoi et al., 2021). In this review, we examine the regeneration of ISCs and the regulation of small intestinal crypt–villus axis cell differentiation in damaged UC mucosa at the molecular level. Our aim was to clarify the pathogenesis of UC and explore new therapeutic strategies.

### ISCs drive intestinal epithelial cell renewal and regeneration

The gut is one of the most complex organs and is critical for the absorption and digestion of nutrients (Gill et al., 2021; Sundaram and Borthakur, 2021). Multiple layers protect the intestine from pathogens (Walther et al., 2019; Wu et al., 2020a; McCauley, 2020). Among these layers, the intestinal epithelium is composed of a single layer of columnar epithelial cells that is the first barrier to pathogens and must remain intact to protect the intestine from infection and toxic agents (Gill et al., 2021). Epithelial cells are classified into two lineages (absorptive and secretory) and are shed into the intestinal lumen every 3–4 days. This rapid cell turnover is due to ISCs that line the bottom of the crypt between the terminally differentiated Paneth cells and epithelial cells that have differentiated along the crypt–villus axis (Filipowska et al., 2022; Singh et al., 2022). After ISCs are damaged, Paneth cells replenish ISCs by providing Wnt ligand and Notch stimulation. Thus, Paneth cells are helper cells that maintain the stem cell microenvironment or niche (Li et al., 2022a; Mead et al., 2022; Ostadmohammadi et al., 2022).

The crypt–villus axis is the basic unit of the intestinal epithelium. A crypt is an area where ISCs and progeny cells proliferate, and a villus is an area where various functional cells differentiate (Iftekhar and Sigal, 2021; Manco et al., 2021). The crypt base is composed of LGR5+ crypt-based columnar (CBC) cells and B-cell-specific Moloney murine leukemia virus Insertion Site 1 (Bmi 1) silent ISCs at the +4 position (Jee et al., 2021; Islam et al., 2022). Studies have shown that LGR5+ (LGR5-labeled ISCs) are generally in an active mitotic state and drive permanent intestinal epithelial cell renewal. However, Bmi 1+ (Bmi 1-labeled ISCs) are in a resting state under normal conditions and exert no substantial effect on intestinal epithelial cell renewal (Atlasy et al., 2019; Ke et al., 2020). However, Bmi 1+ cells are activated upon intestinal epithelial injury and are transformed into LGR5+ stem cells, which are critical for tissue repair after intestinal epithelial injury (Espersen et al., 2015; Li et al., 2019a). ISCs undergo asymmetric division, generating one primary stem cell and one transient amplifying (TA) cell (Milosevic et al., 2018). Primary stem cells undergo self-renewal, while TA cells rapidly migrate to the junction of the crypt–villus axis and differentiate into one of four main functional cell types (Potten et al., 2009; Parry et al., 2013). Among these differentiated epithelial cells, absorption cells account for more than 90% of the population, and they are involved in the induction of nutrient transport and absorption, which is the basis for maintaining the intestinal tract itself and nutrient metabolism in the body (Potten et al., 2009). Goblet cells, which secrete mucin and form a mucus layer, comprise approximately 5% of the intestinal barrier (Cheasley et al., 2011). Intestinal endocrine cells account for approximately 1% of the functional cell population. They secrete cholecystokinin (CCK) and intestinal hormones such as vasoactive intestinal peptide (VIP) and glucagon-like peptide-1 (GLP-1) to regulate the intake, digestion, and absorption of nutrients (McCauley, 2020). Paneth cells (which secrete defensins and lysozymes) account for 10–15% of the cells in each crypt and maintain the activity of ISCs (McCauley, 2020). Thus, Paneth cells and ISCs together maintain the virtuous cycle of the intestinal “ecosystem” (Yokoi et al., 2021). In addition, ISCs differentiate into some rare cell types, including clusters of cells, but their functions remain unknown.

### Molecular mechanism regulating colonic crypt–villus axis cell differentiation

#### Signaling pathways that regulate the proliferation and differentiation of colonic stem cells

Recent evidence indicates that colonic epithelial cells are strictly controlled by multiple signaling pathways, such as the Wnt, Notch, bone morphogenetic protein (BMP) and epidermal growth factor (EGF) signaling pathways, which play important roles in maintaining the self-regeneration, colonization and differentiation of colonic stem cells. Wnt signaling is the main driving force maintaining epithelial cells in the colon, and Wnt is expressed in a gradient along the crypt–villus axis (Ruiz de Sabando et al., 2016). The activity of Notch signaling is the key factor that determines the differentiation of stem cells into differentiating and secretory cells. According to recent studies, the Wnt and Notch signaling pathways coregulate the self-regeneration and differentiation of ISCs (Panda and Biswal, 2019). Mouse LGR5+ stem cells were grown in 3D cultures *in vitro*, and Wnt and Notch signaling pathway activity was modulated by small molecules (Deng et al., 2020). Without the controlling effects of the intestinal tract, stem cells showed new and more differentiated orientations (Kim et al., 2020a). These results show that Wnt and Notch signals maintain the self-colonization of stem cells (Zha et al., 2019). Moreover, inhibition of Wnt signaling and activation of Notch signaling induce the differentiation of intestinal epithelial cells (IECs) (Sasaki et al., 2016). Furthermore, activation of Wnt signaling and suppression of Notch signaling induce Paneth cell differentiation (Ni et al., 2016). In addition, inhibition of Wnt and Notch signaling induced cup-shaped cell differentiation that followed a clear direction (Hirata et al., 2013). However, although the suppression of the Notch signaling pathway led to endothelial secretory cell differentiation, the Wnt signaling pathway exerted little effect on the differentiation of these cells (Ni et al., 2016). Taken together, these studies suggest that the coregulation of signaling pathways is important for the maintenance of the positive functions of colonic stem cells and the balanced differentiation of IECs (Metidji et al., 2018).

### Regulation of transcription factors related to cell differentiation

The links between basal factors and signal transduction and transcription factors must be explored to obtain a better understanding of the mechanisms that differentiate basal factors from signal transduction and transcription factors. Using DNA microarray technology, the heterogeneous expression of genes in mouse crypt–villus axis epithelial cells has been described, and other differentially expressed genes, including those associated with the differentiation and migration of cells, have been identified (Kumar et al., 2018). Motifs related to cell cycle progression and RNA transcription and translation were downregulated during cell maturation (Duchesne et al., 2003). The expression levels of the genes with motifs related to cytoskeletal assembly and lipid uptake that are targeted by the c-Myc oncogene and Cyclin D1 were the lowest in apical villi (Chang et al., 2021). Subsequent reports indicated that the expression of genes encoding homologous transcription factors in the pancreas and duodenum and thyroid hormone receptors was downregulated (Chang et al., 2021). Similarly, the genes expressed at the lowest levels were detected in mouse intestinal cells located at the top of the villus along the crypt–villus axis, and the protein phosphatase (PPP5c) gene was expressed at the highest level at the top of the crypt. Moreover, PPP5c, an important nuclear transcription factor, induced the acquisition of an important phenotype by inducing Pdx1 and Thra expression (Liang et al., 2022a). Through a negative-control experiment, the expression of the target genes SI and Lct was significantly increased in the crypt-villus axis. Based on these results, the differential expression of factors related to enterogenesis regulated the expression of factors related to cell growth and absorption during the differentiation of colonic epithelial cells (Shimakura et al., 2006).

As a specific nuclear transcription factor expressed in IECs, tail-type homeobox transcription factor 2 is widely expressed in distal compartments of villi and mainly regulates the proliferation, differentiation and migration of IECs. Moreover, the expression of the hydrogen/peptide coupling transporter (Pept1) gene in human IECs was regulated by CDX2. Because the Pept1 initiator motif does not contain a canonical T–A sequence region for binding CDX2, Pept1 expression is modulated by an interdomain region connected to the Sp1-binding region in the initiator motif. CDX2 bound to the promoter region of a short-chain lipoic acid transporter, and its mRNA expression was subsequently upregulated. CDX2 is also involved in regulating the expression of several other factors related to IEC proliferation and the intestinal epithelial structure. Studies have shown that CDX2 is activated through cis-acting mucin binding to mucin in the intestinal tract. However, MUC2 is expressed only in goblet cells; therefore, CDX2 plays an important role in goblet cell differentiation (Dalmasso et al., 2008; Coskun et al., 2010). As a member of the calcium-dependent agglutinin superfamily, regenerating factor 4 is a latent activator of the epidermal growth factor receptor (EGFR)/AKT/activator protein-1 signaling pathway. Ectopic activity of this pathway is high in colorectal tumors. CDX2 directly interacts with the initiator motif subregion in Reg IV and can be modulated. Some scholars have analyzed the entire initiator motif related to CDX2 binding and found that captiocytosis and macrophage transporter protein 3 is the underlying target of CDX2. CDX2 and Sp1 coregulate the expression of the ELMO3 gene and thus regulate cell migration (Luo et al., 2022).

The transcription factor GATA belongs to the zinc finger protein family. GATA-4/-5/-6 are expressed in the epidermis of the mature colon, and GATA-4 is present from high to low abundance from the proximal to distal end of the colon. GATA transcription factors inhibit or activate Lct, invertase, GATA-4 and GATA-6, which play important roles in maintaining the structure and differentiation of IECs. Conditional knockout of GATA-4 and GATA-6 in colonic epithelial cells of adult mice led to the proliferation of stem cells in the duodenum and jejunum, a significant decrease in the numbers of intestinal endocrine cells and Paneth cells, and a substantial increase in the number of goblet cells (Nauman and Stanley, 2022). GATA transcription factors inhibit the differentiation of secretory cells through the fine-tuned regulation of the Notch ligand Dll1 (Kholodenko et al., 2016). In addition, CDX2 synergistically regulates the initiation of ISC differentiation and plays a role in regulating the development of the intestinal epithelial structure with GATA-4 and hepatocyte nuclear factor 1α (HNF-1α) (Mao et al., 2022).

### Molecular mechanism regulating nutrient metabolism in colonic epithelial cell renewal and injury repair

Changes in the function of IECs may lead to changes in nutrient metabolism, and the metabolites produced by these cells may also regulate the colonization and development of IECs to a certain extent. The levels of EGF, TGF, amino acids and their derivatives are regulated and controlled by different mechanisms to promote the proliferation and regeneration of IECs after injury (Anzai et al., 2020; Cheng et al., 2022).

#### EGF

**FIGURE 1:**
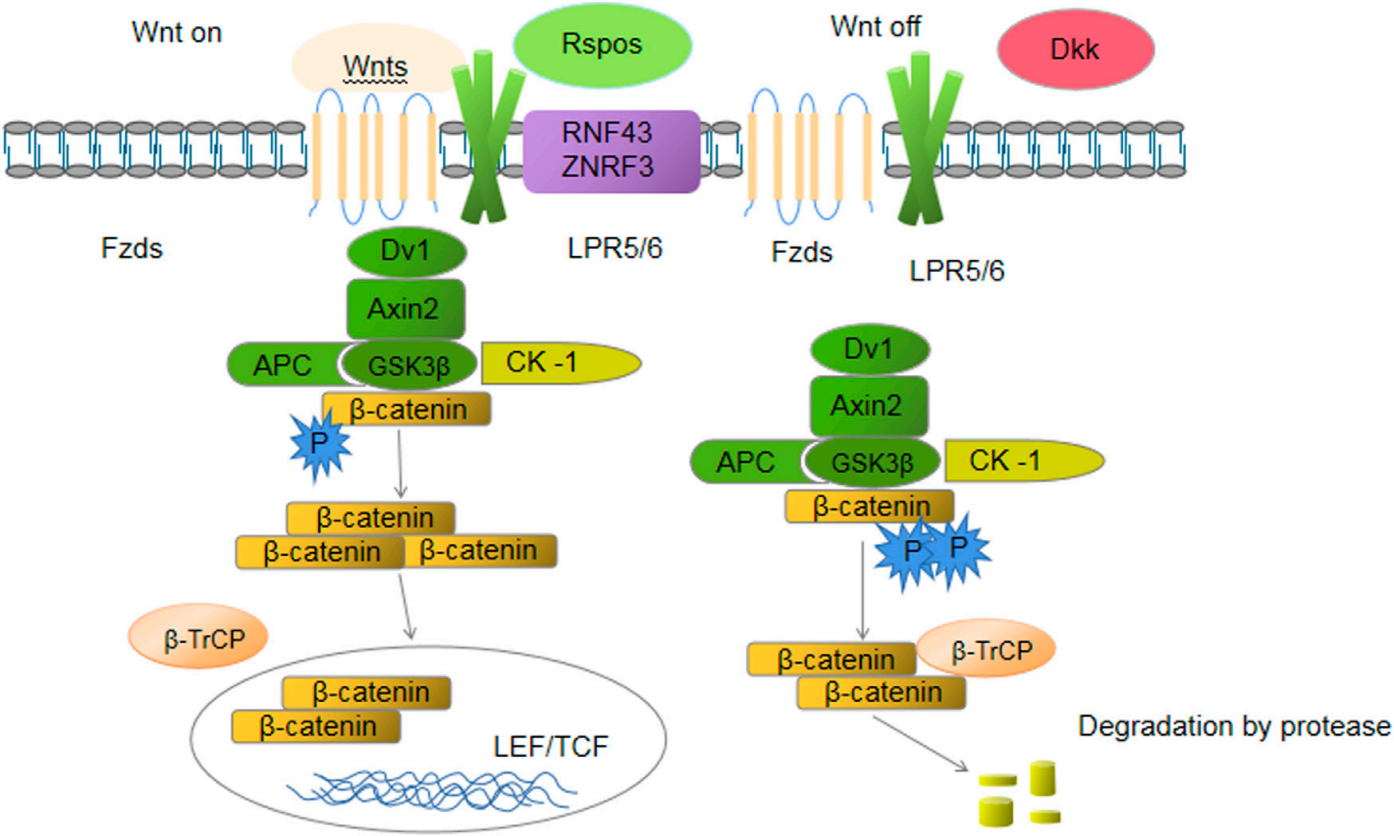
EGF signaling.

EGF is a single-chain small-fragment polypeptide composed of 53 amino acid residues that is abundant in the stomach and intestines. Exogenous EGF promotes the repair of rat small intestine mucous membrane crypt cells after radiation injury, inducing rapid crypt cell proliferation; the results suggested proved that a sufficient quantity of exogenous EGF promotes ISC colonization and repairs damaged IECs (Jijon et al., 2012). Specifically, EGF increases intestinal glutaminase activity, providing intestinal energy and amide nitrogen to promote cell proliferation. Endogenous EGF signaling in crypt–villus axis cells in the intestinal tract promotes the colonization of ISCs and short-lived TA cells. The mechanism of EGF action is triggered after the specific binding to EGFR on the membrane of the cell (Lamubol et al., 2021). EGFR activates other kinases *via* phosphorylation, initiating the activation of a series of downstream signaling pathways (such as PI3K/AKT and RAS/RAF/MEK/ERK) that ultimately activate intracellular transcription factors and regulate downstream gene transcription to modulate the proliferation and differentiation of cells. In crypt cells, the RAS/RAF/MEK/ERK is activated downstream of EGF (Sánchez-de-Diego et al., 2019) (Figure 1).

#### TGF-β

TGF-β is a polypeptide produced throughout the gastrointestinal tract that plays an important role in maintaining the integrity of the epithelial epithelium on the mucosal membrane and regulating IECs. The MAPK and SMAD signaling pathways increase the number of binding proteins on the cell surface and enhance intestinal barrier function during inflammation, and with the gradual attenuation of intestinal mucositis, they increase intestinal permeability. Endogenous TGF-β plays important roles in the colonization of IECs and promotion of cell repair. As an important member of the TGF-β superfamily, BMP participates in regulating cell proliferation, differentiation and apoptosis. It also plays important roles in embryonic development and adult stem cell maintenance. The mechanisms controlling and stabilizing BMP signaling during the development and differentiation of sub-IECs have been elucidated. In the course of early embryonic gut development, the BMP-4 signal is induced in the embryogenic layer, reaching the surface of the embryogenic layer of the inner wall, and cells in the embryogenic layer proliferate from the internode. Subsequently, BMPs mediate the enrichment of villi and the pericrypt mesenchymal mass prior to villus growth (Barruet et al., 2018). In the mature intestinal tract, BMPs mainly control the growth of IECs. Further research has shown that BMP signaling compensates for β-catenin activity that has been directly inhibited by PTEN/PI3K/AKT and SMAD signaling (Ma et al., 2017). Through an antagonistic signaling pathway, the equilibrium between the dynamic proliferation and differentiation of upper cutaneous cells is maintained. The antagonistic cells in the crypt basal layer and peripheral cells secrete Noggin and Chordin to suppress the BMP signal near the crypt (Huang et al., 2018). Therefore, BMP is maintained at a low concentration far from stem cells, promoting the regeneration and continuous colonization of stem cells in the intestinal crypt (Ali et al., 2020).

#### Amino acids and their derivatives

L- Glutamine (Gln) is a nonessential amino acid and the main functional substance of the intestinal tract. Gln deficiency induces the death of IECs and promotes the differentiation of ISCs to form new epithelial cells (Harun-Or-Rashid and Hallböök, 2019). Supplementing cells with Gln promotes cell growth, maintains the integrity of the cell membrane, and enhances the cellular response to hyperoxygenated conditions and mucosal barrier function by strengthening the connections between tight junction proteins (Blachier et al., 2021). In a kinetic model of intestinal resection, a characteristic increase in intestinal mucous membrane growth factors (EGF and IGF-II) was observed (Currò et al., 2017). In addition, EGF and IGF-II promoted the colonization and differentiation of the intestinal epithelium by activating a series of signaling pathways to repair the damaged intestinal epithelium (Zhou et al., 2016). Gln activates MAPK. Gln significantly increases the level of P-ERK when Gln and the ERK signaling pathway are simultaneously activated. Gln also promotes the colonization and differentiation of ISCs by increasing the number of growth factor receptors on the surface of cells and repairing the damaged intestinal epithelium (Söderman et al., 2022).

#### Hippo-YES-associated protein (YAP) pathway

**FIGURE 2:**
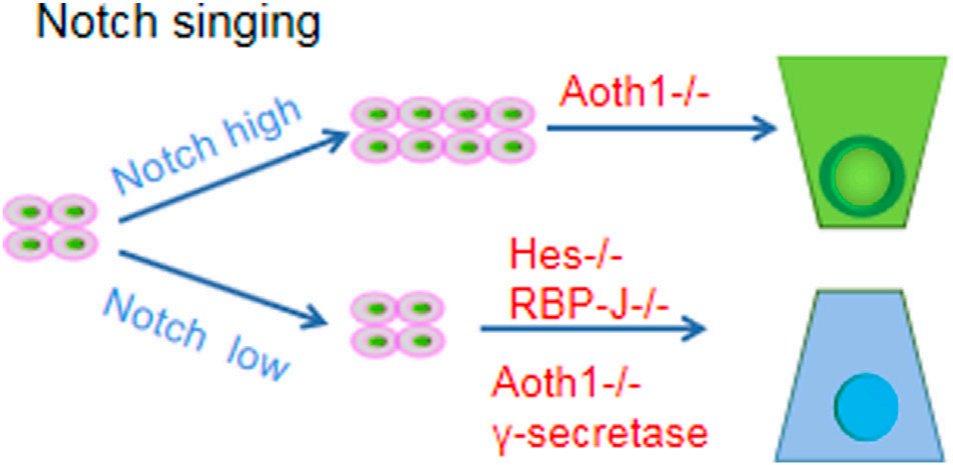
Hippo-YES-associated protein (YAP) pathway.

The Hippo-YAP pathway, also known as the Hippo pathway, is mainly composed of three parts: the upstream regulators MST1/MST2, core factors LATS1/LATS2, and downstream effectors YAP/TAZ (He et al., 2018). In contrast to other pathways, this pathway has no specific receptor or related ligands. The core of the Hippo signaling pathway is a kinase chain that regulates the activity of MST through upstream G-protein-coupled receptor signaling or tyrosine kinase activation and then phosphorylates LATS, leading to the inactivation and degradation of YAP and TAZ in the cytoplasm (Allegra et al., 2021). When MST and LATS are dephosphorylated, YAP and TAZ resume their transcriptional activities, accumulate in the nucleus, and regulate the expression of target genes that promote cell proliferation and cell survival by binding to transcription factors such as TEAD (Park and Kwon, 2018). YAP, a key kinase in the Heme pathway, often plays an important role in the homeostatic regulation of ISCs as a regenerative and proto-oncogene. YAP activity is not only regulated by upstream signaling factors and other signaling pathways but is also sensitive to mechanical signals from neighboring cells and the ECM (Lee et al., 2018a). In the stable state, the Hippo pathway continuously inhibits YAP signaling to maintain the integrity of villi in the crypts. Upon endogenous and exogenous injury, the Hippo pathway activates YAP signaling and participate in the process of LGR5+ ISC proliferation, differentiation and regeneration of IECs to repair intestinal injury (Meng et al., 2016) (Figure 2).

#### Notch signaling pathway

The process of Notch signaling activation requires membrane contact between two cells, where signaling cells express Notch ligands (e.g., Dll1/Dll4) and signal receiving cells express Notch receptors (e.g., Notch1) (Meurette and Mehlen, 2018). After ligand activation, the receptor is hydrolyzed by a series of proteases (particularly the metalloproteinases ADAM10 and γ-secretase), and then the Notch intracellular domain (NICD) translocates to the nucleus to alter the expression of several cofactors (especially RBP-J) (Li et al., 2017). Among them, the Hes family of NICD downstream target genes and Hes and Hey transcription factors are responsible for initiating a broad genetic program after Notch activation that determines the ultimate fate of cells: stem/progenitor cell proliferation or differentiation of cells in the transcriptional expansion zone into absorbing progenitor cells (Siebel and Lendahl, 2017). Notably, lateral inhibition of Notch signaling has been observed in intestinal cells. Cell A expresses the neurogenin-3 gene (event origin - one or more protoneural genes encoding bHLH family transcription factors), stimulates the expression of Delta and terminal differentiation genes such as NeuroD (also belonging to the bHLH family) and drives Cell A differentiation (Christopoulos et al., 2021). At the same time, Delta expressed in Cell A activates Notch ligand on the surface of neighboring Cell B, triggering the expression of another bHLH gene (such as Hes1) in Cell B to inhibit the expression and activation of the Neurogenin-3 gene such that Cell B cannot differentiate (Castro et al., 2021). The presence of lateral inhibition allows cells that have started to differentiate to prevent neighboring cells from differentiating.

In the gut, the Notch signaling pathway has two main roles: *1*) negative regulation to prevent stem cell differentiation and thus maintain the stem cell pool; and *2*) promoting differentiation in one direction and inhibiting differentiation in other directions to regulate the balance between secretory and resorptive cells. In terms of maintaining the stem cell pool, fast-cycling CBC stem cells are characterized by high Notch signaling, which promotes ISC proliferation (Sprinzak and Blacklow, 2021). However, inhibition of Notch signaling by γ-secretase inhibitors, knockdown of the metalloproteinase ADAM10, or inactivation of the ligand Dll1/Dll4 resulted in a loss of CBC stem cells.

**FIGURE 3:**
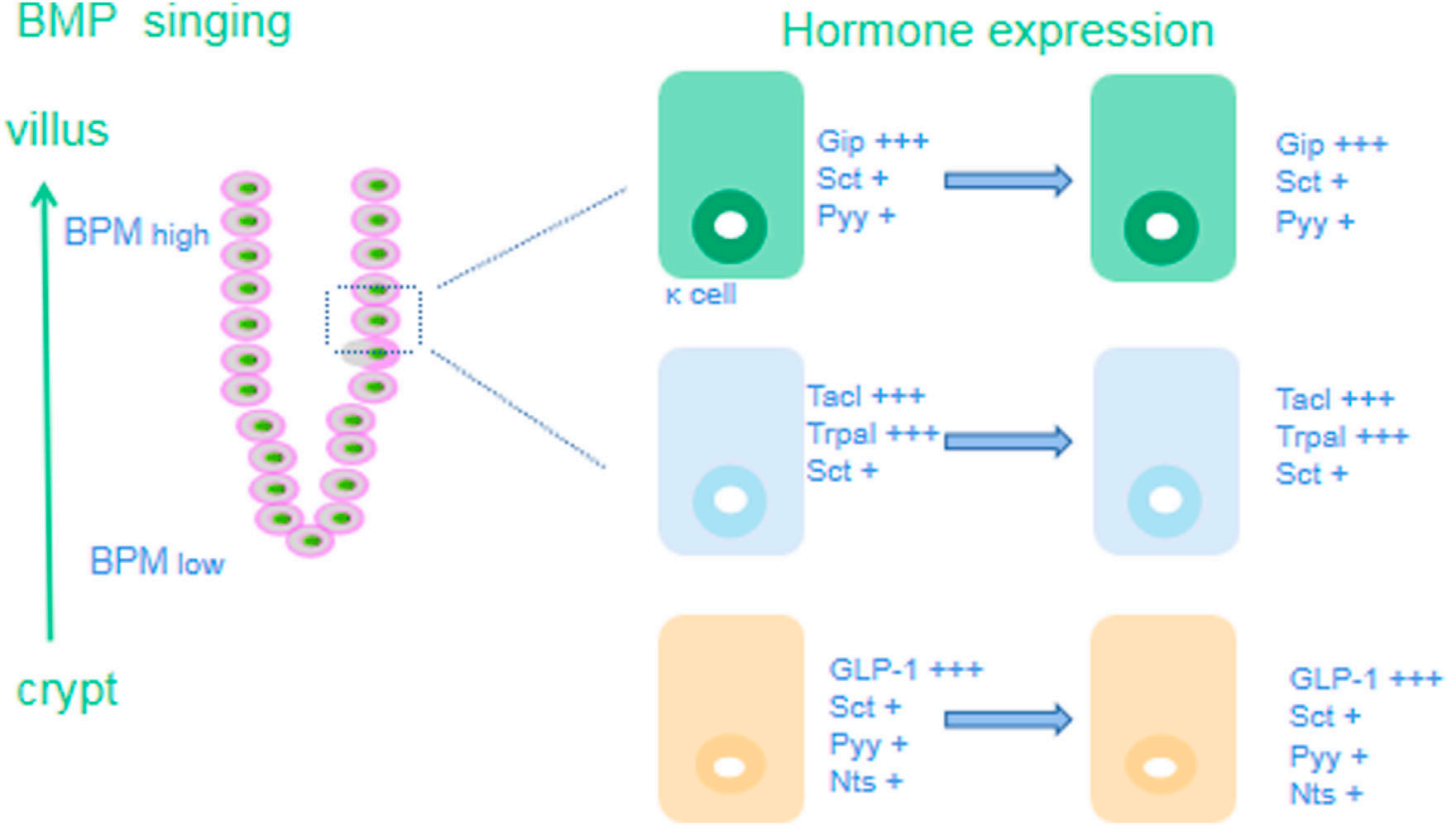
Notch signaling pathway.

In terms of maintaining the balance between resorptive and secretory cells, Notch-low and Notch-high cells are generated to ensure the maintenance and differentiation of stem cells (Golub, 2021). The transcription factor regulating secretory cells is Atoh1 (also known as Math1 or Hath1), and the Hes protein inhibits Atoh1. Therefore, Notch-high cells will differentiate into absorptive cells. For example, Atoh1 knock down will make all secretory cells disappear, while Atoh1 overexpression will transform cells into the secretory phenotype (Guo et al., 2021). For example, the proliferation of ISCs that differentiated into secretory progenitor cells when Notch signaling was inhibited by knocking down the Hes gene or Notch lateral inhibition was removed by knocking down the transcription factor RBP-J or chemically inhibiting γ-secretase (Reicher et al., 2018) (Figure 3).

#### BMP signaling pathway

BMP signaling is mediated by BMP binding to and dimerization of type I and type II BMP receptors (BMPRI and BMPRII), followed by phosphorylation and dimerization of receptor-activated SMAD proteins (Lowery and Rosen, 2018). They then bind the cochaperone SMAD protein (SMAD4) to form a heterotrimeric complex that translocates to the nucleus to regulate target gene expression. Secreted BMP inhibitors, such as chordin-like 1, Gremlin 1, or Gremlin 2, sequester BMP and prevent it from binding to receptors to inhibit BMP signaling (Siebel and Lendahl, 2017).

In the intestinal tract, BMP antagonizes the proliferation signal of the ISC niche and promotes cell differentiation. When BMP signaling is inhibited, ISCs proliferate excessively and exhibit polypoid changes (Ampuja and Kallioniemi, 2018). For example, when BMPR1a, the major receptor for BMPs in IECs, is conditionally deficient in mice, both the stem cell region and the TA region expand and form benign polyps. Similarly, ectopic overexpression of the BMP inhibitors Gremlin1 or Noggin in mouse IECs leads to ectopic crypt overformation and polyp growth. BMP inhibitors such as Gremlin1, Gremlin2, chordin-like1 or Noggin secreted by myofibroblasts and smooth muscle cells under the crypt coregulate BMP expression to form a gradually increasing gradient of BMP concentrations from the bottom of the crypt to the villi as a mechanism to maintain the balance between stem cell proliferation and differentiation (Garcia and Delany, 2021).

**FIGURE 4:**
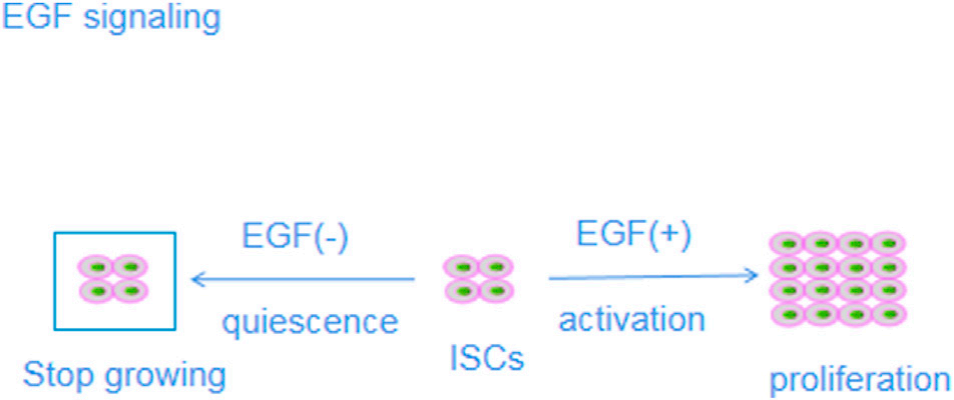
BMP signaling pathway.

The BMP concentration gradient in the gut crypt-villus axis makes EECs subject to increased BMP signaling during their migration from the bottom of the crypt to the villus, which may lead to changes in its hormonal lineage and specific transcription of hormone genes (Yu et al., 2019). EEC subtypes are classified according to the lineage of hormones secreted by the cells: serotonin (5-HT) is produced by enterochromaffin cells (EC); L cells produce glucagon-like peptide-1 (GLP-1), a peptide encoded by the glucagon (Gcg) gene that induces insulin release; and these cells can coexpress Pyy (Lv et al., 2018). K cells mainly secrete gastric inhibitory polypeptide (GIP). D cells secrete somatostatin (Sst). I cells produce CCK, N cells produce neurotensin (Nts), and S cells produce secretin (Sct). Under the influence of the increasing BMP concentration gradient in the crypt-villus axis, the expression of genes specific for various types of cells changes during movement (Wang and Chen, 2018). In the crypt, K cells mainly express high GIP levels but exhibit low expression of Sct and Pyy. When the K cells move from the crypt to the villus, the expression of Sct and Pyy increases compared with the previous cells. During the process of crypt-villus axis movement, the expression of Tac1 and Trpa1 decreases gradually, while the expression of Sct increases gradually (Zylbersztejn et al., 2018). The expression of GLP-1, Sct, Pyy and Nts decreases from high to low during the movement of L cells. The expression of Sct and LAPP in D cells does not change (Sanz et al., 2021). In conclusion, when EECs encounter a high concentration of BMP signaling in villi, most EECs begin to express Sct, and all BMP-activated EECs, except D cells, upregulate Sct expression to varying degrees, whereas L cells increase Pyy and Nts expression. Therefore, in addition to producing specific hormone types, EEC subtypes also express specific genes of other subtypes at high levels during the process of moving to villi, leading to changes in their hormone lineage Figure 4.

### Pathophysiological changes in UC mucosal injury

#### Abnormalities in goblet cells and Paneth cells

IECs include absorptive IECs, goblet cells, Paneth cells and intestinal endocrine cells (Bai et al., 2022). Most cells located near the intestinal lumen are absorptive cells whose main function is metabolism and digestion, while secretory IECs include goblet cells, Paneth cells and intestinal endocrine cells whose role is to digest and maintain the intestinal mucosal barrier (Caioni et al., 2021). Goblet cells are mucus-secreting cells in the epithelium with distinct structural features: the nucleus is located on the basal side, with a large perinuclear region containing rough endoplasmic reticulum, a large Golgi apparatus and condensed vesicles, and distinct granular aggregates composed of mucin (MUC) and other glycoproteins surrounded by a keratin-rich membrane are located at the top (Bai et al., 2022). MUC is the main secretory product of goblet cells and the main component of the mucus layer covering the intestinal surface. By preventing contact between intestinal microorganisms and host epithelial cells and immune cells, MUC effectively prevents an excessive inflammatory response in the intestinal tract and thus plays an important role in maintaining mucosal homeostasis (Li et al., 2022b).

Under normal circumstances, IECs and the mucus layer form a physical barrier protecting against the invasion of pathogenic microorganisms. Goblet cells play an important role in this process because they secrete highly glycosylated mucins, of which MUC2 is the most abundant, as the first line of defense in the intestinal epithelium (Glover et al., 2022). Studies have shown intestinal MUC2 O-glycosylation changes in patients with active UC (Becker et al., 2013). From a pathological perspective, UC lesions are confined to the mucous layer and damage goblet cells to a large extent, leading to bacterial erosion of epithelial cells throughout the mucous layer (Gersemann et al., 2012). The proliferative lesions in individuals with Crohn’s disease primarily form in the submucosa, causing relatively little damage to goblet cells and activating the transcription factors Hath1 and KLF4, thus promoting goblet cell differentiation and increasing MUC secretion (Zheng et al., 2011). Therefore, a change in MUC levels can induce UC, and more importantly, the production of the UC inflammatory response may affect goblet cell differentiation and MUC expression (Yang et al., 2011). These two interactions lead to the occurrence and development of UC (Iwasaki et al., 2011).

In UC, the MUC2 glycosylation sequence is changed, which further aggravates the inflammatory response in individuals with UC (Gersemann et al., 2009). Recent studies using a mouse model deficient in MUC2 highlighted the importance of MUC in maintaining mucosal barrier integrity (Wu et al., 2022). IECs in mice with a MUC2 deficiency lack morphologically distinct goblet cells and show a significant loss of the intestinal epithelial mucosal layer, leading to increased penetrability and the spontaneous development of UC (Kang et al., 2022). In addition, tissue biopsy samples obtained from patients with clinical UC in either the active or remission stage showed indications of significant loss of the intestinal mucosal mucus layer, which indicated reductions in the number of goblet cells and MUC2 secretion, leading to the loss of the intestinal epithelial mucus layer, which is one of the pathological mechanisms of UC (Bets et al., 2022).

Other secretory IECs include Paneth cells, which are characteristic of glands in the small intestine that secrete a variety of antibacterial substances, such as defensin, to inhibit the overproduction of intestinal bacteria. Thus, Paneth cells play an important role in maintaining intestinal homeostasis and participate in innate immunity (Stolzer et al., 2021). Defensin plays an important role in maintaining mucosal balance; thus, researchers have inferred that Paneth cells exhibit bactericidal functions. Moreover, a defensive deficiency or depletion reduces mucosal tolerance, thus leading to intestinal susceptibility (Rana et al., 2021). The Paneth cell distribution increases from the duodenum and jejunum to the ileum. Paneth cells are not detected in the intestinal glands of the large intestine, but the defensins secreted by Paneth cells enter the colon with other contents of the small intestine and participate in the defense of the colon (Wang et al., 2021a). Approximately 75% of the hollow ileal crypts contain Paneth cells, and approximately 50% of the duodenal crypts contain Paneth cells, but Paneth cells are largely absent in colon crypts (Colquhoun et al., 2020). Studies have shown that in subjects with Barrett’s esophagitis and gastritis, a small number of Paneth cells are present in the colon, but the changes in the Paneth cell distribution in individuals with UC are significant, particularly in the regeneration stage (Larabi et al., 2020). Specifically, the number of Paneth cells in the colon is significantly increased, and in some cases, they are present at 300-fold higher than the normal level; the intracellular structure of these cells is also significantly changed in UC. These findings indicate that the protective function of Paneth cells is weakened (Stange, 2017)^,^ (Kurashima and Kiyono, 2017).

Antimicrobial peptides (AMPs) are produced mainly by Paneth cells in the intestinal tract, and among AMPs, α-defensin, which supports the mucus layer, is the most abundant component (Gubatan et al., 2021). In subjects with UC, some tightly regulated genes encode proteins involved in autophagy, granular protein secretion, defensin secretion and endoplasmic reticulum stress, leading to dysfunction of Paneth cells, reduced AMP secretion into the intestinal lumen, and ultimately, increased levels of proinflammatory factors, aggravating the inflammatory response (Simmonds et al., 2014).

Immune abnormalities, including the production of autoantibodies and aberrant cellular immunity, cytokines, oxygen free radicals and nitric oxide, are the main pathological characteristics of UC. A study examining the proportion of Paneth cells in the colons of 10 patients with UC and 10 patients who underwent surgical resection to treat other diseases revealed that the colonic crypts of patients with UC containing more than one Paneth cell accounted for 19.6% of all Paneth cells in intestinal crypts, and in the most extreme case, the proportion reached 30%. No Paneth cells were observed in crypts near the areas of acute inflammation or abscess formation. Another researcher studied 44 patients with UC and found that 41 (93%) presented with Paneth cell metaplasia (PCM) in the colon or rectum (Tanaka et al., 2001). In addition, PCM was positively correlated with crypt distortion and monocyte infiltration but not with crypt atrophy or mucin deficiency, suggesting that the hyperplasia and repair of intestinal mucosa may be important in PCM (Khoramjoo et al., 2022). The intestinal flora of patients with UC is significantly different from that of healthy people. Although no direct evidence has shown that the intestinal flora induces the differentiation of stem cells into Paneth cells through a direct mechanism, we propose that a change in the intestinal flora may cause PCM. The mild Paneth cytogenesis in individuals with UC plays a positive role in maintaining the growth and developmental environment of crypt ISCs and promotes the repair of the injured intestinal mucosa (Khoramjoo et al., 2022). However, because metaplastic cells are derived from crypt stem cells, excessive metaplastic cell differentiation inevitably causes the excessive consumption of crypt stem cells, reducing the number of stem cells available to differentiate into other types of cells and delaying the mucosal repair process. In addition, intestinal mucosa barrier maintenance and other important functions of other types of IECs are reduced. The roles of Notch and Wnt signaling in maintaining the balance between small intestinal stem cell differentiation and Paneth cell differentiation in the colon may mediate the differentiation of colonic stem cells in individuals with UC, which is not a process that occurs in the normal intestine (Molinas et al., 2021). These results suggest that Wnt and Notch activation and Paneth cells may indirectly participate in the pathology of UC. Compared with their levels in normal Paneth cells in the small intestine, neutrophilic alkaline phosphatases (NALPs), namely, NALP1, 7, 8 and 11, were expressed at higher levels in the transformed Paneth cells from patients with UC, corroborating evidence suggesting that Paneth cells may participate in UC pathology (Liu et al., 2019a).

### Role of LGR5+ cells in intestinal mucosal homeostasis

#### Molecular biological characteristics of LGR5

LGR5, also known as GPR49, HG38, FEX, GPR67 and MGC117008, is a seven-transmembrane (TM) protein belonging to the G protein-coupled receptor (GPCR) family. The extracellular region of GPCR family proteins contains 17 leucine-rich repeats that interact with glycoprotein ligands, a 7-alpha helix transmembrane region that is highly conserved, and N-terminal and C-terminal tails enriched in cysteine residues (Kuo et al., 2018). No physiological ligand was initially identified for LGR5; therefore, it was called an orphan receptor. The ligand of LGR5 is an R-spondin (Rspo) protein, and the binding of LGR5 enhances Wnt/β-catenin signaling and plays multiple important roles in stem cell growth. The extracellular N-terminus of the LGR5 protein binds to the Rspo protein, but evidence suggests that binding does not trigger G-protein signaling (Carmon et al., 2017). However, LGR5 is associated with GPCRs, such as thyroid-stimulating hormone (TSH), follicle-stimulating hormone (FSH), and luteinizing hormone (LH) receptors. These hormone receptors are rich in leucine residues in the extracellular N-terminal domain and contain nine repeating units that bind glycoprotein hormones (Snyder et al., 2017). A DNA hybridization analysis showed that the LGR5 gene was highly conserved throughout mammalian evolution. The human LGR5 gene has been mapped to chromosome 12q22-23 and shown to have a full-length cDNA sequence consisting of 4208 bp, including 22 exons and 21 introns. Transcription of the cDNA produces eight different mRNAs, five of which are generated through alternative splicing (Jee et al., 2021). The open reading frame encodes a polypeptide chain consisting of 907 amino acid residues, including 21 signal peptides, 540 outer functional regions, 263 transmembrane regions, and 83 C-terminal tail regions. Compared with the few tissues expressing glycoprotein hormones such as TSH, LGR5 is expressed in a variety of tissues, such as skeletal muscle, gastrointestinal tract, hair follicles, the brain, spinal cord, mammary glands, eyes and reproductive organs (Boonekamp et al., 2021).

LGR5 is a recognized marker of stem cells. LGR5 us also expressed in cancer stem cells (CSCs) and possesses extensive stem-like pluripotency and self-renewal properties (Mei et al., 2020). In 2007, a class of crypt-base columnar cells (CBCs) with positive expression of the LGR5 gene was identified in the +1 ∼ +3 position between Paneth cells at the base of crypts. These cells are highly proliferative and radiation-sensitive. At the same time, ISCs are considered true ISCs, which are characterized by high plasticity and multilineage differentiation of progeny and long-term self-renewal. Experimental studies have shown that a single LGR5+ ISC isolated from the gut is sufficient to form endosomes, a three-dimensional culture system, and can generate all epithelial cell types, replenish the entire crypt-villus axis of the gut, and play a very important role in the stable regeneration of the entire gut (Fang et al., 2022)^,^ (Chen et al., 2022a). The imbalance of LGR5+ ISC function may induce inflammatory bowel disease, intestinal tumors, polyps, adenoma, bowel cancer, short bowel syndrome and other related intestinal diseases. In addition, under certain circumstances, some terminally differentiated cells, including Paneth cells and enteroendocrine cells, also have a certain degree of plasticity. These cells can also re-enter the ecological niche and dedifferentiate into LGR5+ ISCs to replenish their numbers and meet the requirements for stem cell proliferation.

#### Wnt/β-catenin signaling pathway

The classical Wnt signaling pathway is characterized by the accumulation and transcription of β-catenin in the nucleus; hence, it is also called the Wnt/β-catenin signaling pathway (Jee et al., 2021). The Wnt/β-catenin signaling pathway is highly conserved during evolution and participates in complex interactions with other signaling pathways, such as Notch and BMP signaling, to drive the orderly self-renewal and differentiation of ISCs, ensure crypt cell proliferation and maintain intestinal homeostasis (Qi et al., 2022). Wnt/β-catenin signaling components include Wnt ligands, frizzleds (Fzds), the β-catenin degradation complex, β-catenin, lymphoid enhancer factor/T-cell factor proteins (LEF/TCFs), and various target genes (Davidson, 2021). Wnts in the intestinal stem cell microenvironment are mainly secreted by Paneth and mesenchymal cells. Fzds and their coreceptors, low-density lipoprotein (LDL) receptor-associated proteins (LRPs; e.g., LRP5 and LRP6), signal to the disheveled protein (Dvl). Activated Dvl reduces glycogen synthase activity through adenomatous polyposis coli (APC), axis inhibition protein (Axin) and glycogen synthase (glycogen synthase) kinase 3β, GSK 3β), phosphoprotein phosphatase 2A (PP2A) and casein kinase-1 (CK-1). By inhibiting the phosphorylation of β-catenin, β-catenin accumulates in the cytoplasm and translocates to the nucleus, eventually binding to LEF/TCFs to replace transcriptional inhibitors on the promoter of target genes, thereby regulating the transcription of downstream target genes and stimulating signal transduction cascades in one or more cells. The target genes of Wnt include c-Myc, Cyclin D1 and LGR5, among which c-Myc and Cyclin D1 promote the proliferation of IECs, and LGR5 enhances the activity of ISCs (Sahebdel et al., 2022). In contrast, in the absence of Wnt ligands, β-catenin is phosphorylated by degradation complexes and is recognized and degraded by ubiquitin proteasomes.

In addition, mesenchymal cells and Paneth cells secrete Rspos that regulate Wnt/β-catenin signaling and promote intestinal development and regeneration (Giebel et al., 2021). The Rspo family includes four secretory growth factors (Rspo1-4) that are essential for stimulating the proliferation and differentiation of ISCs. *In vitro*, Rspos support the survival, expansion and budding of ISCs, and a lack of Rspo1 leads to crypt cell and ISC apoptosis. Enhanced Wnt/β-catenin signaling induced by Rspos is mediated mainly through the formation of a complex with ubiquitin ligase and LGR family factors (Raslan and Yoon, 2019). Induced automatic ubiquitination and clearance of zinc and ring finger 3/ring finger 43 (ZNRF3/RNF43) inhibit ZNRF3/RNF43 activity and cause the Wnt receptor to accumulate on the cell membrane. When Rspo is inactivated, ZNRF3/RNF43 induces Fzd and LRP degradation through the ubiquitin-dependent protein degradation pathway, enhancing the binding sensitivity of Wnts (Lebensohn and Rohatgi, 2018).

Two types of extracellular antagonists target Wnt/β-catenin signaling (Larabi et al., 2020). In one type, the antagonist competitively binds a Wnt ligand, along with Fzds, to inhibit Wnt signaling. These antagonists include secreted Frizzled-related protein (sFRP) and Wnt inhibitory factor (WIF) (Xie et al., 2021). Another type of Wnt receptor competitively binds to Wnt ligands to exert inhibitory effects; these antagonists include members of the Wnt inhibitor (Dickkopf, DKK) family (Sunkara et al., 2021).

#### LGR5 and colonic stem cells

Regeneration of the colonic mucosal epithelium is maintained by ISCs located between Paneth cells at the base of the colonic crypt (Baharudin et al., 2020). ISCs are characterized by high expression of LGR5 and are very sensitive to changes in Wnt signaling. ISC progeny comprise TA cells, the population of which is increased by several cycles of mitosis and migrates up the crypt (Cai et al., 2022). TA cells undergo cell cycle arrest and terminal differentiation when they leave the crypt and approach the intestinal lumen. Recent studies have analyzed biomarkers to determine the precise location of these stem cells in the intestinal crypt (Chen et al., 2022b). The most notable of these markers is LGR5, which indicates ISC fragmentation. LGR5+ ISCs are long-lived, proliferate continuously, and can differentiate into different types of terminally differentiated epithelial cells, including absorptive cells, goblet cells, intestinal endocrine cells, and Paneth cells, while maintaining their stem cell status (Chiang et al., 2022). Single LGR5+ crypt stem cells form organoid structures *in vitro* (Wang et al., 2022). LGR5+ colonic stem cells were transplanted into GFP+ cells on the surface of the damaged colon of mice to assess their survival after transplantation. Four weeks after transplantation, LGR5+ colonic stem cells formed a single layer of epithelial cells and a functional and histologically normal intestinal crypt with self-renewal ability (Bomidi et al., 2021). Colonic stem cells proliferated *in vitro* to form tissue for transplantation (Eggington et al., 2022). In addition, we found that intestinal crypt homeostasis depends on the symmetric division of LGR5+ stem cells by tracing LGR5+ stem cells using multicolor Cre recombinase receptors (Lee et al., 2022). The study was based on pedigree tracing of LGR5+ stem cell activity in the mouse colon and revealed that LGR5+ cells, representing 5%–10% of the stem cell population, produced additional LGR5+ cells as along with all types of inflammatory cells and accelerated inflammatory responses (Leystra et al., 2021). In most cases, intestinal inflammation is driven by mutations in Wnt signaling pathways (Wu et al., 2021). In mice, LGR5+ cells were more likely to cause intestinal inflammation than other intestinal epithelial cell populations with mutations in the Wnt signaling pathway. These results suggest that LGR5+ stem cells initiate colon inflammation. Therefore, LGR5 is a marker of colonic stem cells (Girish et al., 2021).

#### LGR5 is a specific marker of ISCs

LGR5 expression has been assessed to identify ISCs. LGR5 is specifically expressed in CBCs, and LGR5+ CBCs present in the gastric antrum, small intestine and colon differentiate into different types of cells (Jee et al., 2021). In addition, LGR5+ cells in villi in the colonic crypts regenerate various types of IECs (Sheng et al., 2020). Therefore, LGR5 is considered a specific marker of colonic ISCs (Ayyaz et al., 2019). LGR5+ cells exhibit the activity of colonic stem cells and have the potential for self-renewal and multipotent differentiation (Rodríguez-Colman et al., 2017).

#### LGR5+ ISCs promote intestinal mucosal regeneration

LGR5+ ISCs were injected into the intestinal mucosa of mice with UC induced by sodium dextran sulfate (DSS) by enema. The intestinal mucosa injury in these mice was treatable, and the regeneration and repair of the intestinal epithelium was promoted (Cedeno et al., 2017). These results indicate that LGR5+ ISCs play important roles in the regeneration and repair of the injured intestinal mucosa (Beumer and Clevers, 2016).

After intestinal mucosal injury caused by UC is established, colonic stem cells located at the base of the crypt continuously proliferate, and the progeny migrate along the crypt–villus axis and ultimately reach the top of the crypt, where they differentiate into mature colonic epithelial cells (Li et al., 2016). However, this differentiation is inhibited or terminated by mucosal inflammation. LGR5+ cells, however, regenerate colon epithelium (Basak et al., 2014). Therefore, transplantation or amplification of ISCs may be therapeutic options for UC (Kim et al., 2012). Recent studies have shown that the transplantation of LGR5+ ISCs attenuates intestinal mucosal injury induced by DSS in UC mice (Hu et al., 2021). In addition, some scholars have indicated that expanding colonic stem cells *in vitro* and then autologously transplanting them into injured sites of the colon may ameliorate UC. These results and assumptions may provide researchers with new ideas and research directions for the treatment of UC in the clinic (Zullo et al., 2021).

In summary, LGR5+ ISCs generate IECs through self-renewal and multipotential differentiation to promote regeneration and repair of the intestinal mucosa. Different signaling pathways exert different regulatory effects on ISCs (Zullo et al., 2021). The roles of the Wnt and Notch pathways in the regulation of ISCs have been described. The Wnt signaling pathway is the main driving force for ISC proliferation and mucosal renewal, and the Notch signaling pathway promotes the differentiation of ISCs (Geng et al., 2018).

### Role of the Wnt signaling pathway in UC mucosal injury

#### LGR5 and the Wnt/β-catenin pathway

Studies of UC have revealed that LGR5 is a stem cell marker in many self-renewing tissues, including the intestinal tract and hair follicles, and is a target gene in the Wnt pathway closely related to the occurrence of UC. Wnt signaling induces Axin binding to phosphorylated LRP, displacing the degradation complex (Qu et al., 2015). β-Catenin stably resides in the cytoplasm, translocates into the nucleus, and binds to TCRF/LEF to form a transcription complex, which induces the transcription of LGR5 and Gastrin target genes (Xu et al., 2022). The LGR5 family modulates the involvement of Rspos in the classical Wnt signaling pathway, and LGR proteins have been shown to tightly bind Rspos. Even a small amount of Wnt stimulation enhances Wnt signaling and contribute to UC occurrence (Dong et al., 2022). Studies on the interaction between LGR5 and Wnt receptors suggest that LGR5 is part of the Wnt signaling complex and enhances Wnt/β-catenin signaling at the membrane level. Immunohistochemistry revealed the expression of LGR5 and β-catenin in tissues from 102 patients with UC (Yu et al., 2021). Moreover, LGR5 was positively correlated with the expression of β-catenin, suggesting that the involvement of LGR5 in the occurrence of UC may be mediated by the Wnt/β-catenin pathway. After eliminating the APC gene in lGR5-CRE+ mouse ISCs, transformed ISCs remained at the bottom of the crypt for 3-5, and β-catenin was expressed at high levels in the nucleus, suggesting that LGR5 promoted β-catenin expression and activated the Wnt signaling pathway (Wang et al., 2021b). The mechanism by which LGR5 enhances Wnt signaling remains unclear. However, some scholars have suggested an opposing view, suggesting that LGR5 does not enhance Wnt signaling but exerts a negative regulatory effect. When the expression of LGR5 was downregulated through RNA interference technology, LGR5 expression was decreased, exerting an increased inflammatory effect. In contrast, LGR5 overexpression resulted in increased cell adhesion, decreased colony formation and reduced inflammatory effects (Fountzilas et al., 2021). Expression profiles also showed that LGR5-knockout cells exhibited increased Wnt signaling and upregulated expression of genes related to the epithelial-mesenchymal transition (EMT), in contrast to the expression profile of cells with high LGR5 expression (Zhang et al., 2021b).

In the absence of Wnt signaling, the degradation complex composed of the tumor suppressor genes APC and Axin, tyrosine kinase i (TK ⅰ) and glycogen synthase kinase 3 (GSK3) targets β-catenin, leading to phosphorylation and proteasome degradation and ultimately resulting in the inactivation of the Wnt signaling pathway (Contreras et al., 2020). The Wnt/β-catenin signaling pathway is activated by the binding of Wnt glycoproteins to their transmembrane coreceptors, namely, Fzd protein and LRP5/6 (Nong et al., 2021). The WNT-Fzd-LRP5/6 complex triggers signal transduction, causing β-catenin to translocate to the nucleus and bind to different transcription factors in a complex (TCF4/LEF), initiating Wnt target gene transcription (Dupasquier et al., 2019).

Studies have shown that LGR5 is the downstream target gene in the most important β-catenin signaling pathway (Lybrand et al., 2019). In addition, LGR5 binds Rspo1. Rspos constitute a group of secretory proteins that enhance Wnt/β-catenin signaling pathway activity and promote ISC proliferation. LGR5 binds tightly to Rspo1 by inducing LRP6 phosphorylation, thus activating the Wnt/β-catenin signaling pathway (Sheng et al., 2020). After conditional deletion of LGR5 in the intestinal epithelium, the number of ISCs and Wnt target gene expression decrease rapidly. These results suggest that the reduction or depletion of LGR5 caused by UC mucosal injury reduces the activity of the Wnt/β-catenin signaling pathway, which is not conducive to the regeneration of the intestinal epithelium. In summary, LFR5 binds to Rspo1 to activate the Wnt/β-catenin signaling pathway in conjunction with Wnt ligand-Fzd-LRP5/6 complex formation (Lee et al., 2018b).

Wnt signaling is closely related to the regulation of ISC proliferation. ISCs express LGR proteins and Fzd. When the ligand Wnt and Fzd bind Rspo and LGRs, the destruction complex is inhibited, followed by β-catenin accumulation. Groucho enters the nucleus to displace this complex and then binds to TCFs to regulate the expression of genes by driving transcription, thereby regulating the proliferation of stem cells. In the absence of Rspo and Wnt, Fzd is degraded after ubiquitination mediated by the RNF43 or ZNRF3 protein, and the destruction of the complex will induce β-catenin degradation, resulting in its inability to bind to TCF and ultimately leading to the loss of LGR5+ stem cells. Activation of Wnt signaling leads to ISC expansion (Lee et al., 2018b).

Wnt concentration gradients regulate stem cell proliferation. Paneth cells produce the ligand Wnt3, which decreases as stem cells begin to differentiate and leave the stem cell region, creating a concentration gradient of Wnt3 as they move along the crypt-villus axis and creating a dynamic negative feedback loop. When stem cells proliferate faster, a large number of cells move toward the villi to reduce the Wnt concentration in the stem cell region and then reduce the proliferation of stem cells and the output of cells in the crypt. When stem cells are lost, their proliferation is slow, the Wnt concentration gradient is shortened, and the Wnt level at the bottom of the crypt is increased, which is conducive to the symmetrical differentiation of stem cells or the dedifferentiation of progenitor cells into stem cells to achieve the purpose of stem cell proliferation.

#### The Wnt/β-catenin signaling pathway promotes ISC proliferation and maintains intestinal epithelial homeostasis

The regeneration of the intestinal epithelium depends on the rapid response of ISCs, which are amplified and replenished by adjacent crypt stem cells in cases of injury and driven by LGR5+ stem cells to quickly complete repair (Liu et al., 2019b). Studies have shown that LGR5 expression is stimulated when the intestinal epithelium is exposed to a single stressor, while LGR5 expression is decreased when the intestinal epithelium is exposed to multiple stressors, indicating that ISCs are sensitive to external stimuli and that a slight injury may accelerate the proliferation and differentiation of injury-repairing stem cells (Al-Hakeim et al., 2021). Sustained or intense stimulation might result in the loss of a large number of stem cells, decreased regeneration of the intestinal epithelium, and severe villus atrophy (Brown et al., 2022). Since Wnt/β-catenin signaling regulates LGR5 expression, appropriate upregulation of Wnt/β-catenin signaling increases LGR5+ stem cell activity and promotes crypt–villus axis reconstruction (Li et al., 2019b).

The Wnt/β-catenin signaling pathway is essential for ISC proliferation. Recently, an experiment with a human colonic crypt model showed that the classical Wnt pathway and the inhibitory TGFβ/BMP pathway promote the regeneration of the human colonic epithelium and repair injured intestinal mucosa (Xian et al., 2017). However, other studies revealed that intestinal crypt destruction and IEC proliferation were significantly inhibited in transgenic mice expressing the Wnt pathway inhibitor DKK1 (Saha et al., 2016). In contrast, ISCs exposed to Rspo, a Wnt pathway agonist, entered a highly proliferative state (Yum et al., 2016). These results further suggest that the Wnt/β-catenin signaling pathway is the main driver of ISC proliferation (Becker and LeBleu, 2018).

Another important link between the Wnt pathway and intestinal mucosal homeostasis is the bidirectional regulation of MUC expression and Wnt pathway activation (Zieba et al., 2022). MUC2 interacts with β-catenin *via* a C-terminal protein domain, which promotes the nuclear translocation and aggregation of β-catenin and contributes to the activation of the Wnt pathway (Liang et al., 2022b). β-Catenin also directly affects the transcription of MUC2 and other MUC genes. This molecular mechanism suggests that the reduced expression of MUC2 in UC is related not only to a reduction in the number of goblet cells but also to the abnormal regulation of the Wnt pathway (Fortini et al., 2014).

**FIGURE 5:**
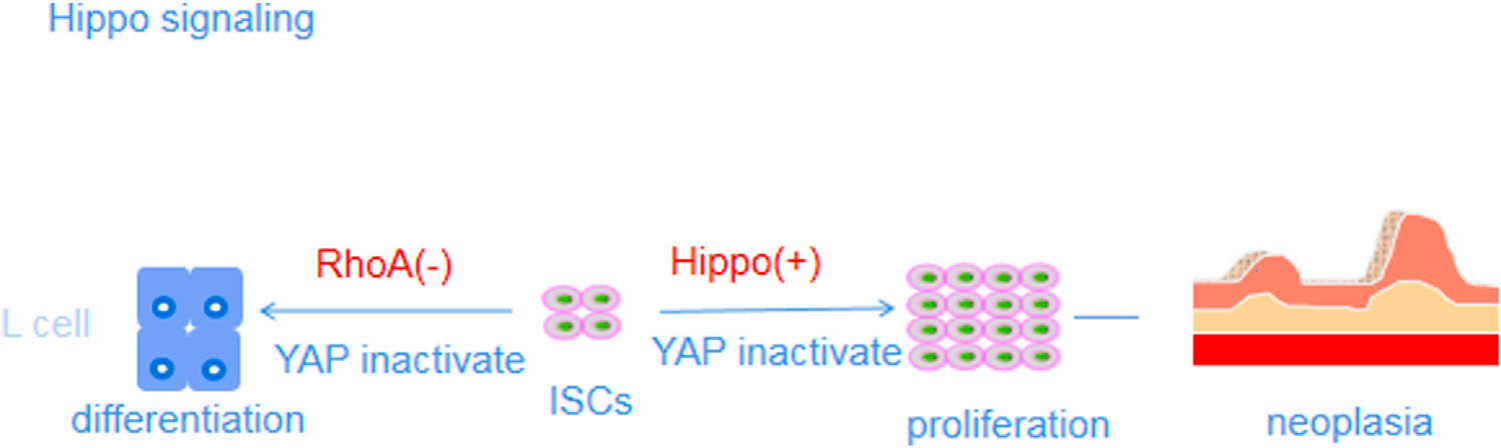
Mechanisms of action of Wnt/β-catenin signaling pathways.

In summary, these studies suggest that the Wnt pathway is the main driving force for ISC proliferation and that it maintains intestinal homeostasis. In clinical applications, inhibitors and agonists of the Wnt pathway may be therapeutic targets to regulate the overactivation and inhibition of the Wnt pathway, respectively, under pathological conditions to restore normal Wnt pathway activity and maintain a normal ISC proliferation rate as a method to establish intestinal epithelial homeostasis (Zhou et al., 2020) (Figure 5).

#### Notch signaling promotes the differentiation of ISCs

The Notch signaling pathway consists of receptors, ligands and DNA-binding proteins. Notch signaling is activated by the interaction of Notch receptors with ligands on two adjacent cells (Fujisawa et al., 2021). The binding of the receptor to the ligand results in a conformational change in the receptor, which exposes the extracellular protease cleavage site of the receptor for subsequent gamma-secretase-mediated proteolysis, releasing the NICD (Pistol et al., 2021). The NICD translocates to the nucleus, where it binds to the transcriptional repressor RBP-J and induces the transcription of Hes and other genes (Yang et al., 2008).

Notch signaling regulates the differentiation of ISCs into absorptive cell types. The conditional deletion of RBP-J and gamma-secretase inhibitor treatment to block NICD release resulted in the differentiation of crypt ISCs into secretory cells (Won et al., 2022). Similar results were observed in young mice deficient in Hes1, a Notch target gene, which led to an increase in the number of secretory cells and a decrease in the number of absorptive cells (Ho et al., 2022). However, due to early death of the mice, the effect of Hes1 deficiency on adult mice is uncertain (Tsai et al., 2022). The Notch pathway negatively regulates the differentiation of secretory cells after the inhibition of Math1, a target gene in the Notch pathway, promoting RBP-J expression (Bahuguna et al., 2021). In addition, loss of Math1 causes the abnormal differentiation of mucosal goblet cells, leading to loss of the colonic mucus layer, which induces UC (Shi et al., 2021). Notch signaling pathway activity mediated by Dll1 and Dll4 maintains the balance between intestinal mucosal inflammatory injury and repair, and the number of goblet cells is increased in Dll1-specific knockout mice, suggesting that Dll1 exerts a particularly significant regulatory effect on crypt ISC function (Chen et al., 2021a).

Based on these results, Math1 promotes the differentiation of ISCs into secretory cell types, while Hes1 promotes the differentiation of ISCs into absorptive cell types (Kurokawa et al., 2020). Both of these proteins jointly modulate the Notch signaling pathway to regulate the differentiation of LGR5+ ISCs and maintain intestinal homeostasis, a process in which the ligand Dll1 may play a major role (Chen et al., 2021b). The Notch pathway target genes Hes1 and Math1, as well as the ligand Dll1, may be new therapeutic targets for the treatment of UC, and their actions may provide a theoretical basis for further research (Kwak et al., 2021).

#### Interaction between the Wnt and Notch pathways

Although the Wnt and Notch pathways have been confirmed to play important roles in intestinal homeostasis, the specific mechanisms by which the two pathways interact to regulate ISC activity and directed differentiation remain unclear (Lee et al., 2021). According to recent studies, Notch-blocking antibodies increase the level of the ligand Wnt3, decrease the NICD level in ISCs and TA cells, and induce the differentiation of ISCs into secretory cell types. Shortly after Notch pathway blockade, the inhibition of the Wnt pathway is released, its activity is upregulated, and LGR5+ ISCs differentiate into secretory cell types, reducing LGR5+ ISC activity and attenuating their depletion. These results suggest that the transformation of secretory cells after Notch blockade may be caused by the overactivation of the Wnt pathway following Wnt3 upregulation (Kim et al., 2020b). Notch expression and ISC activity were restored after an anti-LPR6 antibody was used to reduce Wnt output, and the number of Math1+ secreting cell progenitors was also reduced (Takahashi et al., 2020). Therefore, an appropriate level of Wnt pathway activity is required to simultaneously maintain the proliferation and differentiation of ISCs. The Notch pathway inhibits the output of Wnt signaling while maintaining ISC activity and differentiation, and an antagonistic interaction characterizes the relationship between these two pathways, which enables their joint maintenance of intestinal homeostasis (Baulies et al., 2020). In the pathological condition of UC, the balance of the Wnt and Notch signaling pathways may be disrupted, which may cause intestinal dysfunction, affect the proliferation and differentiation of ISCs, and prevent normal repair of intestinal mucosal injury (Bohin et al., 2020).

Recently, the Wnt and Notch signaling pathways and their interactions were reported to regulate the proliferation and differentiation of ISCs. However, researchers have not clearly determined whether other pathways also play a role in these processes, and further experiments are needed (Khaminets et al., 2020).

In conclusion, intestinal mucosal injury is an important pathological change in individuals with UC (Wu et al., 2020b; Gao et al., 2020). The self-renewal and sustained regeneration of IECs after injury are related to ISCs in the crypt base, which maintain intestinal epithelial homeostasis. During homeostasis, ISCs produce daughter cells (or TA cells) that differentiate into functional cells along the crypt–villus axis (Ballweg et al., 2018). In addition, LGR5+ ISCs are intricately regulated by the Wnt/β-catenin signaling pathway and Notch signaling pathway (Andriatsilavo et al., 2018; Hayakawa et al., 2019). The Wnt/β-catenin and Notch signaling pathways jointly maintain the function of LGR5+ ISCs. More importantly, ISCs located at the base of the crypt proliferate and differentiate into mature IECs through the intricate regulation of these signaling pathways to repair the damaged intestinal mucosa (Bae et al., 2018; Jaeckel et al., 2018). Therefore, transplantation of LGR5+ ISCs, where they can affect multitargeted combined signaling pathways, may be a new approach for the treatment of UC (Yin and Xi, 2018).
